# Development and validation of a chemotherapy tolerance prediction model for Chinese multiple myeloma patients: The TM frailty score

**DOI:** 10.3389/fonc.2023.1103687

**Published:** 2023-01-20

**Authors:** Yadong Chen, Jingli Gu, Beihui Huang, Junru Liu, Xiaozhe Li, Juan Li

**Affiliations:** Department of Hematology, The First Affiliated Hospital of Sun Yat-sen University, Guangzhou, China

**Keywords:** multiple myeloma, frailty, comprehensive geriatric assessment (CGA), timed up and go test, MNA-SF

## Abstract

**Objective:**

The physical fitness of older individuals is heterogeneous, making it difficult to know their chemotherapy tolerance. The toxicities may offset the benefits of anti-myeloma therapy in frail patients. The accurate evaluation of frailty status before chemotherapy is essential. We aimed to explore the applicability of the IMWG GA and develop a new frailty screening tool more suitable for Chinese MM patients.

**Cases and methods:**

We performed the IMWG GA and the full CGA in 167 MM patients and validated the applicability of the IMWG GA to chemotherapy and prognosis. The CGA domains were screened for their predictive value to improve IMWG GA and develop new frailty screening tools.

**Results:**

The results showed that the IMWG GA had limitations in distinguishing the risk of grade ≥3 adverse events (AEs) between fit and int-fit patients. Of the CGA domains, TUG and MNA-SF were independent prognostic factors for grade ≥3 AEs and OS and further stratified the risk of grade ≥3 AEs in the IMWG GA int-fit subgroup (P< 0.05). We combined TUG and MNA-SF to construct the TM frailty score. The frail subgroup had a higher proportion of adverse outcomes, a higher hazard ratio (HR) in Cox regression and a higher Harrell’s C-index for distinguishing the risk of grade ≥3 AEs and OS than the IMWG GA frail subgroup.

**Conclusion:**

The TM frailty score is more suitable than the IMWG GA for evaluating chemotherapy tolerance and prognosis in the Chinese population.

## 1 Introduction

MM is more common in the older population and, to date, is an incurable hematologic malignancy. The widespread use of novel agents improves overall survival (OS) (1), but the improvement of older individuals is not as good as that of young individuals. The main reason is that the chemotherapy tolerance of older individuals is lower; additionally, these individuals cannot tolerate high-intensity chemotherapy to obtain better remission depth, and death from severe chemotherapy toxicity has been observed in this population (2). Furthermore, even among older patients of the same age, physical fitness is heterogeneous. This heterogeneity makes it difficult for therapists to predict the chemotherapy intensity that will match the patient’s chemotherapy tolerance (3, 4). This is more likely to lead to severe chemotherapy-related adverse effects or inadequate treatment and difficulty achieving optimal clinical outcomes.

The accurate evaluation of frailty status is essential before administering chemotherapy. There are several specialized frailty assessment tools for patients with MM, such as the IMWG GA (5), Revised Frailty Algorithm (6), Revised Myeloma Comorbidity Index (7), and Mayo Frailty Model (8), among which IMWG GA is the most widely used frailty assessment tool in the literature for patients with myeloma. The IMWG GA comprises age and three additional domains, the Charlson comorbidity index (CCI), activities of daily living (ADL) and instrumental activities of daily living (IADL), stratified patients into three subgroups of fit, Int-fit and frail (5). IMWG GA evaluation well stratified the risks of nonhematological grade ≥3 AEs and prognosis in older MM patients (5). However, the development and validation of the IMWG GA were based on Western MM populations, and its applicability in the Chinese population remains to be further explored.

The CGA is a multidimensional evidence-based assessment of functional status, comorbidities, nutritional status, psychosocial status, and other domains (9). CGA impairment is highly consistent with patients’ functional independence, hospital length of stay, and the risk of mortality and has been proven to be a powerful predictor of chemotherapy-related AEs for older individuals with cancer (10). Currently, the American Society of Clinical Oncology (11) and International Society of Geriatric Oncology (12) guidelines recommend the CGA for evaluating the physical fitness of older patients with cancer to predict their treatment tolerability.

In this study, we aimed to explore the applicability of the IMWG GA in Chinese MM patients and screened the valuable domains of CGA to further improve the predictive value of IMWG GA. We used the findings to develop a new frailty assessment tool more suitable for the Chinese MM population.

## 2 Methods

### 2.1 Patients and methods

This study was a single-center retrospective study. Ethical approval was obtained from the ethics committee of the First Affiliated Hospital of Sun Yat-sen University. The recruited population comprised consecutive patients newly diagnosed with MM from June 2019 to September 2021. All patients provided informed consent. All patients were diagnosed as symptomatic MM according to the 2014 IMWG criteria (13). There were no age restrictions for inclusion in our study. The included patients were those who were willing and able to comply with the study regulations alone or with the assistance of their family members. Patients had to agree to undergo medically supervised chemotherapy, report adverse events after chemotherapy and accept subsequent efficacy evaluation. Patients who refused chemotherapy, did not receive chemotherapy or did not cooperate with the study team in getting chemotherapy on time were excluded. Patients who were discharged from the hospital or treated in other medical institutions and patients whose adverse events, disease progression, survival and other information after chemotherapy could not be collected during follow-up visits or via telephone were also excluded.

### 2.2 The IMWG GA and CGA

All enrolled patients underwent the IMWG GA and full CGA before chemotherapy. The assessment included questions on age, ADL (14), IADL (15), and the CCI (16). According to the methods and evaluation criteria defined in the original text of the IMWG GA (5), the patients with a final total score of 0 were classified as fit; those with a score of 1 were classified as int-fit; and those with scores of 2-5 were classified as frail.

The participants underwent the CGA using standardized methods administered by trained clinicians. Eight health domains considered essential to patients’ overall health status were evaluated: (1) The Timed Up and Go (TUG) test of the CGA was used to assess the level of functional status, as it not only measures mobility but also is an objective, quantifiable indicator of an individual’s physical fitness and functional capacity (17). According to the TUG distribution characteristics of our patients and those in previous reports, we used TUG <8 s, 8-12 s, and >12 s as the cutoff values, where TUG>8 s indicated functional status impairment. (2) Comorbidities were assessed using the CCI (16). (3) Nutritional status was evaluated using the Mini Nutritional Assessment-Short Form (MNA-SF) (18). The total weighted MNA-SF score ranged from 0 to 14, with a score of 8 to 11 indicating a risk of malnutrition and a score of 7 or lower indicating malnutrition. (4) Polypharmacy was defined as the concurrent prescription of ≥ 5 long-term medications (19). (5) Cognitive impairment was evaluated using the Mini Cognitive Scale (Mini-Cog) (range 0-5) (20). (6) The Hospital Anxiety and Depression Scale (HADS) (21) was used to assess psychological status, where a HADS score of more than 7 points (range 0-21) indicated psychological impairment. (7) The Medical Outcomes Study–Social Support Survey (MOS-SSS) (22) was used to assess social support. It has a total of 20 items and a score that ranges from 20 to 100 points, where higher scores indicate better perceived social support. (8) Geriatric syndrome was defined as a self-reported history of more than two age-related symptoms, such as hearing impairment, vision impairment, sleeping disorder, and falls (23).

### 2.3 Follow-up

The induction regimen of transplant-eligible patients was a three-drug regimen of bortezomib, doxorubicin, and dexamethasone (PAD). After 4-6 cycles of induction therapy followed by autologous stem cell transplantation (ASCT) and maintenance treatment. Patients who were ineligible for ASCT were treated with eight cycles of a two-drug regimen induction therapy, mainly bortezomib and dexamethasone (VD) or lenalidomide and dexamethasone (RD), followed by maintenance treatment. The maintenance treatment used single-agent immunomodulators (thalidomide, lenalidomide) or VD.

The follow-up observational assessments after chemotherapy included the following: 1) adverse reactions to chemotherapy, where all chemotherapy-related toxicities were evaluated according to the Common Terminology Criteria for Adverse Events 5.0 (CTCAE_V 5.0, 2017), and only grade ≥3 AEs were recorded; 2) treatment discontinuation defined as missed chemotherapy, delay of more than two weeks, or suspension of treatment; and 3) time to progression (TTP) and OS, where TTP and OS were defined as the time from observation to progression or death. Disease progression was defined according to the IMWG criteria (24).

In the follow-up observation of grade ≥3 AEs and treatment discontinuations, the observation time of patients eligible for ASCT was from induction therapy until hematopoietic stem cell mobilization (approximately 4-6 chemotherapy cycles). Patients who were ineligible for ASCT underwent 8 chemotherapy cycles. The deadline for the calculation of TTP and OS was September 30, 2021.

### 2.4 Statistical analysis

Continuous variables are presented as medians (interquartile ranges (IQRs)), and categorical variables are summarized according to each category’s number and percentage of patients. Adverse reactions related to chemotherapy were summarized according to the number of events, and descriptive statistics were used to describe the baseline characteristics of the patients and the AE results. The Kaplan–Meier (KM) method was used to analyze the correlation between grade ≥3 AEs, treatment discontinuation, TTP, and OS. The log-rank test was used to compare the curves. The Cox proportional hazards model was used to perform multivariate analysis. A receiver operating characteristic (ROC) curve was performed and Areas under the curve (AUCs), sensitivity, and specificity were reported to reflect recognition ability of the functional status tools, and the differences in the AUC values were compared using the Delong test. The diagnostic ability of these indicators was evaluated by calculating Harrell’s C-index; a C-index between 0.50 and 0.70 indicated low accuracy, and a C-index > 0.70 indicated good performance. Most statistical analyses were performed with SPSS (SPSS Statistics, Version 26.0.; IBM, Armonk, NY, USA). The C-index was computed using R, version 2.14.2. P<0.05 indicated that the difference was statistically significant.

## 3 Results

### 3.1 The applicability of the IMWG GA in Chinese MM patients

#### 3.1.1 Patient characteristics and IMWG GA assessment results

A total of 176 patients met the inclusion criteria, 5 refused chemotherapy, and 4 did not cooperate with data collection. Thus, 167 patients were included in the final analysis. The baseline disease characteristics of the patients are shown in **Table 1**. According to the IMWG GA, 62 (37.1%) patients were classified as fit, 53 (31.7%) as int-fit, and 52 (31.1%) as frail.

**Table 1:** Baseline disease characteristics of the patients (N=167).

	N (%)	Median (IQR)
Sex		–
Male	95 (56.89)	
Female	72 (43.11)	
Age		59 (52, 66)
<65	117 (70.06)	
65-75	39 (23.35)	
76-80	5 (2.99)	
>80	6 (3.59)	
Type of myeloma		–
IgG	74 (44.31)	
IgA	27 (16.17)	
IgD	23 (13.77)	
Light chain	43 (25.75)	
Creatinine, mg/dL		0.84 (0.70, 1.22)
<2	141 (84.43)	
≥2	26 (15.57)	
R-ISS stage		
I	30 (17.96)	
II	100 (59.88)	
III	37 (22.16)	
ADL		6 (6, 6)
>4	140 (83.83)	
≤4	27 (16.17)	
IADL		3 (3, 8)
>5	76 (45.51)	
≤5	91 (54.49)	
CCI		–
≤1	120 (71.86)	
>1	47 (28.14)	

IQR, Interquartile range.

#### 3.1.2 The IMWG GA successfully predicted grade ≥3 AEs but not treatment discontinuation

In total, 99 patients experienced 246 grade ≥3 AEs. The grade ≥3 AEs were classified as hematological AEs and nonhematological AEs (**Supplementary Table 1**). Grade ≥3 AEs were recorded in 28 (45.2%) fit, 29 (54.7%) int-fit, and 42 (80.8%) frail patients.

**Figure 1:**
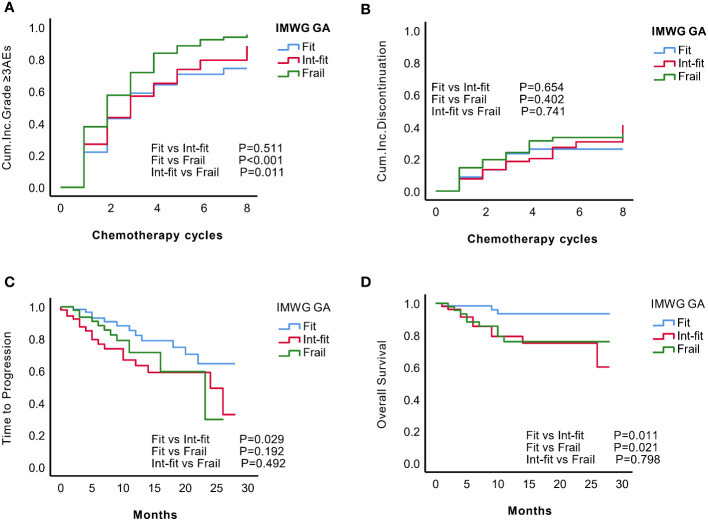
KM analysis of grade ≥3 AEs, treatment discontinuation, time to progression (TTP) and overall survival (OS) according to the IMWG GA subgroups. According to the IMWG GA evaluations, the patients were classified as fit, int-fit, or frail. The three subgroups were compared according to the **(A)** cumulative incidence of grade ≥3 AEs; **(B)** cumulative incidence of treatment discontinuation; **(C)** TTP; and **(D)** OS.

The risk of grade ≥3 AEs in the IMWG GA frail subgroup was significantly increased after chemotherapy and was well distinguished from the risk in the fit subgroup (log-rank test, P<0.001; HR 1.59, 95% CI 1.13-2.23, P=0.008) and int-fit subgroup (log-rank test, P=0.011; HR 1.45, 95% CI 1.04-2.32, P=0.028), whereas there was no significant difference between the fit and int-fit subgroups (log-rank test, P=0.511; HR 1. 09, 95% CI 0.75-1.58, P=0.599) (**Figure 1A**, **Table 2**).

**Table 2:** Multivariate analysis of the impact of the IMWG GA results on the incidence of grade ≥3 AEs, treatment discontinuation, TTP, and OS.

	Grade ≥3 AEs		Treatment discontinuation		TTP		OS	
	HR (95% CI)	*P*	HR (95% CI)	*P*	HR (95% CI)	*P*	HR (95% CI)	*P*
Fit	1	–	1	–	1	–	1	–
Int-fit	1.09 (0.75-1.58)	0.599	1.03 (0.56-1.90)	0.933	1.90 (0.90-4.02)	0.092	3.69 (1.01-13.52)	0.049
Frail	1.59 (1.13-2.23)	0.008	1.16 (0.68-2.03)	0.593	1.29 (0.57-2.90)	0.544	3.08 (0.81-11.72)	0.098

Adjusted for age and R-ISS stage.

In total, 56 patients experienced 74 treatment discontinuation events, including 13 (20.1%) fit patients, 18 (34.0%) int-fit patients, and 25 frail (48.1%) patients. No statistically significant difference in the risk of treatment discontinuation between IMWG GA subgroups was observed (log-rank test, P>0.05, **Figure 1B**).

#### 3.1.3 The IMWG GA fit subgroup had better OS but not TTP

After a median follow-up of 12 months (IQR 6-21), 22 (13.2%) deaths were observed: 16 (9.58%) patients died of disease progression, including 2 (3.2%) in the fit subgroup, 9 (16.9%) in the int-fit subgroup, and 5 (9.61%) in the frail subgroup. Five (3.0%) patients died of adverse chemotherapy reactions, including 1 (1.9%) in the int-fit subgroup and 3 (5.77%) in the frail subgroup. One patient died of other reasons.

The 1-year progression-free rate was 82.2% in the fit subgroup, 63.3% in the int-fit subgroup, and 71.5% in the frail subgroup. The int-fit subgroup was associated with a short TTP by the KM curve (log-rank test, P=0.029; **Figure 1C**), but this lost statistical significance in multivariate analysis (HR 1.90, 95% CI 0.90-4.02, p=0.092) (**Table 2**).

The 1-year OS rate was 93.5% in the fit subgroup, 79.3% in the int-fit subgroup, and 76.0% in the frail subgroup. The patients in the int-fit (HR 3.69, 95% CI 1.01-13.52, P=0.049) had a shorter OS time than those in the fit subgroup (log-rank test, P<0.05; **Figure 1D**), while no significant difference was observed between the fit and frail subgroups (HR 3.08, 95% CI 0.81-11.72, P=0.098). There was no significant difference in OS between the int-fit and the frail subgroups (HR 0.84, 95% CI 0.34-2.08, P=0.703).

### 3.2 Screening for valuable CGA domains to improve the predictive ability of IMWG GA

#### 3.2.1 Of the CGA domains, TUG and MNA-SF are independent risk factors for grade ≥3 AEs and OS

We performed a full CGA on most patients to screen for the valuable CGA domains to improve the predictive ability of IMWG GA. A total of 135 (80.84%) patients completed the full CGA (**Table 3**).

**Table 3:** Characteristics of 8 domains of the CGA (N=135).

CGA Domain	Classification	N (%)
TUG	<8 s	68 (50.37)
	8-12 s	32 (23.70)
	>12 s	35 (25.93)
CCI	≤1	94 (69.63)
	>1	41 (30.37)
MNA-SF	12-14	57 (42.22)
	8-11	53 (39.26)
	0-7	25 (18.52)
Polypharmacy	<5	96 (71.11)
	≥5	39 (28.89)
Mini-Cog	4-5	101 (74.81)
	0-3	24 (17.78)
HADS	≤7	89 (65.93)
	>7	46 (34.07)
MOS-SSS	≥60	69 (51.11)
	<60	66 (48.89)
Geriatric syndromes	≤2	98 (72.59)
	>2	37 (27.41)

The relationship between the full CGA domains and adverse clinical outcomes was investigated by multivariate Cox regression and adjusted for age and R-ISS stage (**Supplementary Table 2**.). The results showed that functional status (TUG) and nutritional status (MNA-SF) were independent risk factors for grade ≥3 AEs (TUG: HR 1.39, 95% CI 1.17-1.64, P=0.001; MNA-SF: HR 1.39, 95% CI 1.14-1.67, P=0.001) and OS (TUG: HR 2.11, 95% CI 1.26-3.58, P=0.006; MNA-SF: HR 1.85, 95% CI 1.01-3.40, P=0.046). Only the Min-Cog test was a statistically meaningful indicator (HR 0.56, 95% CI 0.32-1.00, P=0.049) for treatment discontinuation. No significant associations were observed between any CGA domain and TTP (P>0.05).

#### 3.2.2 TUG can better identify chemotherapy tolerance and poor prognosis than ADL and IADL

We further explored whether TUG has advantages over ADL and IADL in reflecting chemotherapy tolerance and prognosis. In the ROC curves, TUG performed well in identifying grade ≥3 AEs over 4 cycles of induction chemotherapy, with an area under the curve (AUC) of 0.717, which was significantly better than that of ADL (AUC=0.600, ΔAUC=0.117; Z-test, Z=3.282, P=0.001) and an expected trend better than that of IADL (AUC=0.640, ΔAUC=0.077; Z-test, Z=1.70, P=0.074) (**Table 4**) (**Supplementary Table 3**). Even combining ADL and IADL, their AUC improved to 0.669, but they still did not perform as well as TUG (ΔAUC=0.048; Z - test, Z = 1.164, P = 0.244). No predictive value was observed for disease progression within one year (AUC 0.509-0.544, P>0.05). In the ROC analysis for death within 1 year, the TUG had a better AUC (0.629, p<0.05), but the AUC for this parameter was not significantly different than that for other functional status tools.

**Table 4:** Diagnostic value of functional status tools for AEs ≥ grade 3, treatment discontinuation, disease progression and death.

	AEs ≥ grade 3			Treatment discontinuation			Progression within 1 year			Death within 1 year		
	Sens.	Spec.	AUC	Sens.	Spec.	AUC	Sens.	Spec.	AUC	Sens.	Spec.	AUC
TUG	67.0%	76.3%	0.717*	68.6%	58.3%	0.635*	50.0%	53.4%	0.517	70.0%	55.8%	0.629*
ADL	25.3%	94.7%	0.600*	28.6%	87.1%	0.578	17.6%	84.2%	0.509	20.0%	84.4%	0.522
IADL	64.8%	63.2%	0.640*	68.6%	52.3%	0.604*	58.8%	49.6%	0.542	70.0%	50.3%	0.602*
ADL+IADL	64.8%	63.2%	0.669*	68.6%	52.3%	0.628*	58.8%	49.6%	0.544	70.0%	50.3%	0.605

Sens., sensitivity; Spec., specificity.

*P<0.05.

In Cox multivariate analysis adjusted for age, and R-ISS stage, TUG (HR 2.11, 95% CI 1.26-3.58, P=0.006) was confirmed to be independently associated with OS. However, the ADL and IADL subgroups showed no statistical significance (ADL: HR 1.28, 95% CI 0.43-3.75, P=0.650; IADL: HR 1.89, 95% CI 0.77-4.67, P=0.167) (**Supplementary Table 4**).

#### 3.2.3 TUG and MNA-SF can be used to further distinguish the risk of grade ≥3 AEs in the IMWG GA int-fit subgroup

Based on the above results, TUG and MNA-SF were potentially valuable CGA domains for improving the discriminatory ability of IMWG GA. We found that 22.6%, 56.6%, and 76.9% of the patients in the IMWG GA fit, int-fit, and frail subgroups exhibited TUG impairment (TUG >8 s), respectively, and 37.1%, 66.0%, and 88.5% of them were at risk of malnutrition (MNA-SF ≤11).

**Figure 2:**
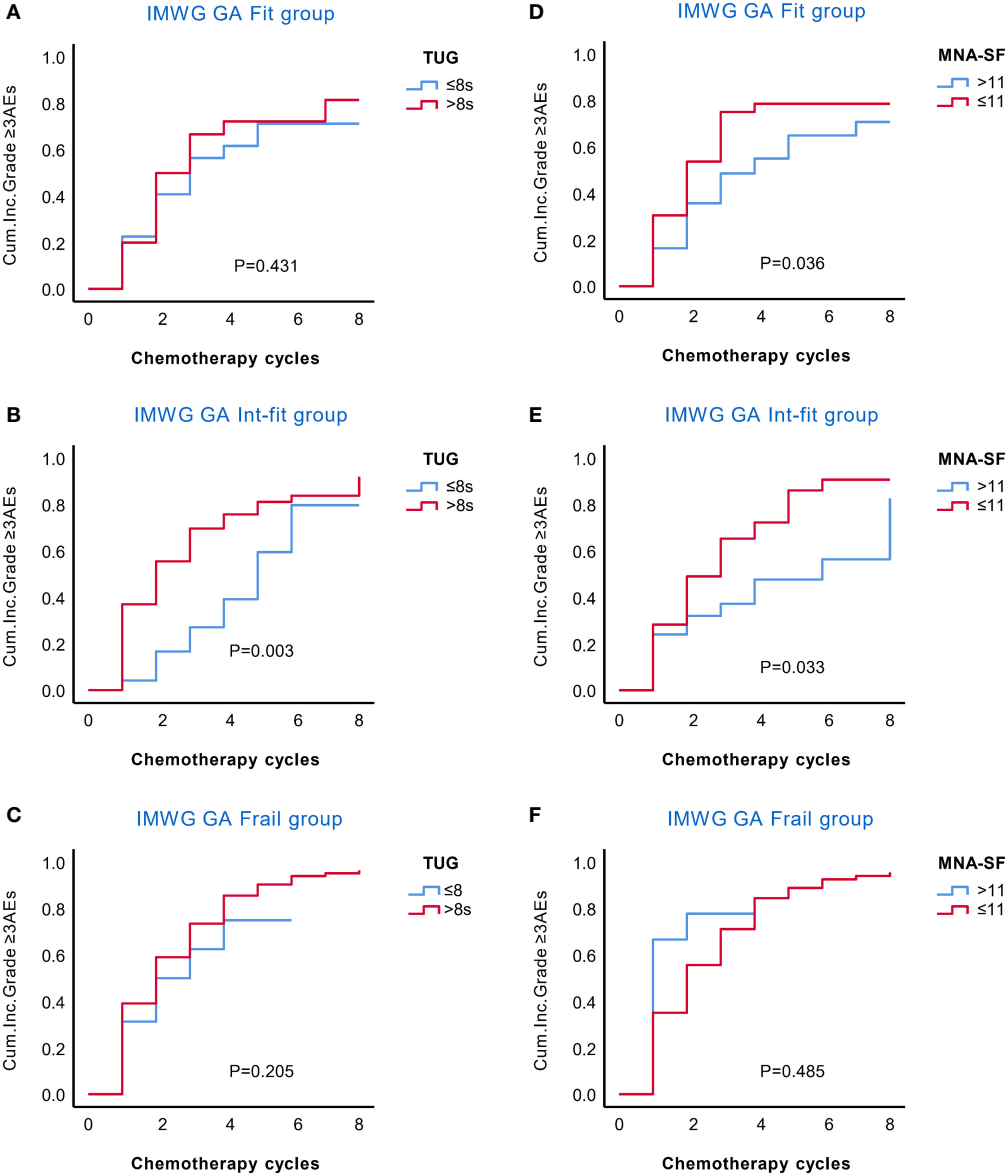
KM analysis of the occurrence of grade ≥3 AEs in the IMWG GA subgroups according to the TUG and MNA-SF scores. The rate of grade ≥3 AEs in the IMWG GA subgroups was stratified by the TUG **(A–C)** and MNA-SF **(D–F)** scores. TUG only stratified the risk of grade ≥3 AEs in the int-fit subgroup **(B)**, but no significant association was found in the fit and frail subgroups **(A, C)**. The MNA-SF stratified the risk of grade ≥3 AEs both in the fit and int-fit subgroups **(D, E)**, but no significant association was found in the frail subgroup **(F)**.

We further explored whether the TUG and MNA-SF scores could stratify the risk of AEs in IMWG GA subgroups. In the KM curves (**Figure 2**), TUG only stratified the risk of grade ≥3 AEs in the int-fit subgroup (log-rank test, P = 0.003), but no significant association was found in the fit and frail subgroups (log-rank test, P =0.431 and 0.205, respectively). MNA-SF stratified the risk of grade ≥3 AEs both in the fit and int-fit subgroups (log-rank test, P =0.036 and 0.033, respectively), but no significant association was found in the frail subgroup (log-rank test, P =0.485).

### 3.3 Adding the MNA-SF did not satisfactorily improve the frailty evaluation performance of the IMWG GA

**Figure 3:**
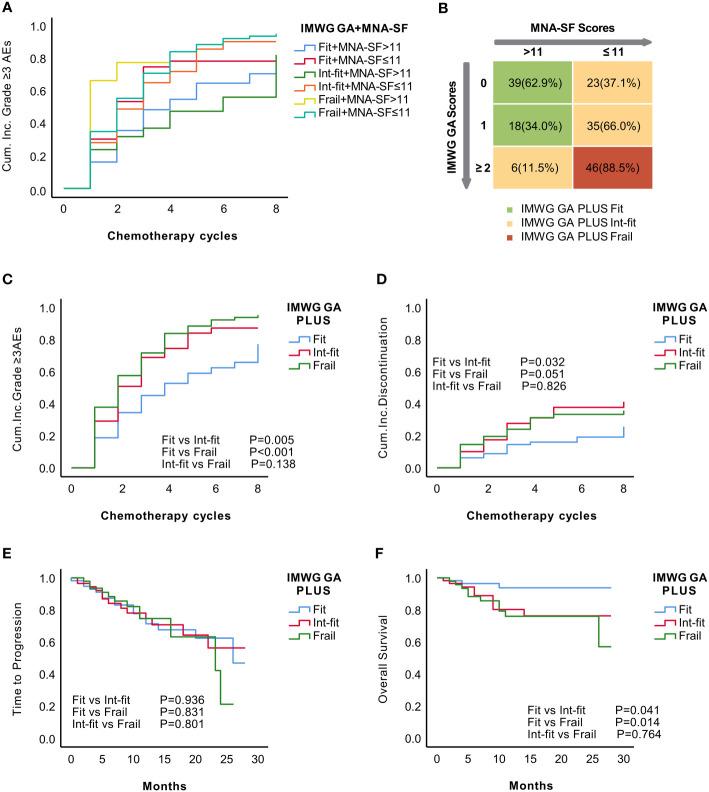
Establishment of the IMWG GA PLUS frailty score as the combination of the IMWG GA score and the MNA-SF score of the CGA. KM analysis for the occurrence of grade ≥3 AEs in recategorized subgroups according to the combination of the IMWG GA and MNA-SF **(A)**. Distribution of MNA-SF score in the IMWG GA subgroups and the final grouping using the IMWG GA PLUS frailty score **(B)**. The KM curve showed that IMWG GA PLUS significantly distinguished the risk of grade ≥3 AEs **(C)** and tended to distinguish the risk of treatment discontinuation (p=0.051) between patients in the fit and frail subgroups **(D)**. There was no significant difference in TTP **(E)**. The KM curve for OS of IMWG GA PLUS seemed similar to that of the IMWG GA **(F)**.

Given that the MNA-SF well stratified the IMWG GA subgroups in the risk of grade ≥3 AEs and nutritional status evaluation was not included in the IMWG GA, we decided to combine the MNA-SF with the IMWG GA to develop a new frailty score, the IMWG GA PLUS frailty score. We divided the patients into 6 subgroups according to their MNA-SF and IMWG GA subgroups and drew a KM curve as a basis for frailty stratification (**Figures 3A, B**).

The patients were stratified into three subgroups according to the KM curves: (1) IMWG GA-fit+ MNA-SF score >11 and IMWG GA-int-fit+ MNA-SF score >11 in the IMWG GA PLUS fit subgroup; (2) IMWG GA-fit+ MNA-SF score ≤11, IMWG GA-int-fit+ MNA-SF score ≤11, and IMWG GA-frail+ MNA-SF score >11 in the IMWG GA PLUS int-fit subgroup; and (3) IMWG GA-frail+ MNA-SF score ≤11 in the IMWG GA PLUS frail subgroup. The final IMWG GA PLUS groupings are shown in **Figure 3B**. After stratification by IMWG GA PLUS, there were 57 (34.13%), 64 (38.32%), and 46 (27.55%) patients in the fit, int-fit, and frail subgroups, respectively.

The IMWG GA PLUS fit subgroup had a significantly different risk of grade ≥3 AEs than patients in the int-fit (log-rank test, P=0.005) and frail subgroups (log-rank test, P<0.001) had, while the risk between the int-fit and frail subgroups (log-rank test, p=0.138; **Figure 3C**) was not well distinguished. Regarding the risk of treatment discontinuation, the fit subgroup appeared to be distinguishable from the int-fit and frail subgroups (log-rank test, P=0.032 and 0.051, respectively) (**Figure 3D**). No differences in TTP were found across the IMWG GA PLUS subgroups (**Figure 3E**). For OS, the KM curve of IMWG GA PLUS seemed similar to that of the IMWG GA: patients in the fit subgroup were distinguishable from those in the int-fit and frail subgroups (log-rank test, P=0.041 and 0.014, respectively) (**Figure 3F**).

Harrell’s concordance index (C-index) values of IMWG GA PLUS were 0.701 (P<0.001) for grade ≥3 AEs and 0.656 (<0.001) for treatment discontinuation, showing a better predictive ability than the IMWG GA (C-index values of 0.662 and 0.636, respectively; P<0.05) (**Table 5**). However, the C-index of IMWG GA PLUS for OS was 0.618 (P=0.020), which was lower than that of the IMWG GA (0.631, P=0.029). Our results suggest that IMWG GA PLUS was not predictive of TTP (P=0.926).

**Table 5:** Harrell’s concordance index (C-index) in different frailty assessment tools.

Frailty model	Grade ≥3 AEs		Treatment discontinuation		TTP		OS	
	C-index	*P*	C-index	*P*	C-index	*P*	C-index	*P*
TM frailty score	0.741	<0.001	0.690	<0.001	0.545	0.397	0.702	0.001
IMWG GA	0.662	<0.001	0.636	0.002	0.559	0.379	0.631	0.029
IMWG GA PLUS	0.701	<0.001	0.656	<0.001	0.483	0.926	0.618	0.020

### 3.4 Development and validation of a new frailty prediction model

#### 3.4.1 Including selected CGA indicators to develop a novel frailty scoring system

Adding only MNA SF to IMWG GA did not satisfactorily improve the ability to identify frailty. Given that TUG can better identify the chemotherapy tolerance and poor prognosis, we decided to replace ADL and IADL with TUG. We included the TUG and MNA-SF scores along with the original factors in the IMWG GA (age, CCI) to establish a multivariate Cox regional model for predicting grade ≥3 AEs. We randomly selected 83 patients as the training set, and the remaining patients were the validation set. There was no significant difference between these two subsets (**Supplementary Table 5**). The results of the multivariate Cox analysis in the training set are shown in **Table 6**. Finally, a scoring system was developed consisting of TUG, MNA-SF, named the TM frailty score. Patients were divided into 3 subgroups according to the TM frailty score: 0 = fit; 1 = int-fit; 2-4 = frail.

**Table 6:** Multivariate Cox proportional hazards model for grade ≥3 AEs.

Variable		HR (95% CI)	*P*	β	Score
TUG	<8	1	0.054		0
	8-12	1.33 (0.91-1.95)	0.144	0.285	1
	>12	1.59 (1.09-2.30)	0.016	0.461	2
MNA-SF	12-14	1	0.134		0
	8-11	1.37 (0.97-1.94)	0.072	0.318	1
	0-7	1.48 (0.96-2.23)	0.074	0.393	2
Age	≤65	1	0.394		–
	66-75	1.06 (0.73-1.55)	0.180	-0.220	–
	>75	1.11 (0.59-2.08)	0.457	-0.158	–
CCI	≤1	1	0.808		–
	>1	0.961 (0.64-1.33)		-0.070	–
R-ISS stage	I	1	0.773		–
	II	1.17 (0.75-1.81)	0.493	0.154	–
	III	1.18 (0.71-1.96)	0.516	0.168	–

#### 3.4.2 Validation of the TM frailty score for distinguishing patients at risk of adverse clinical outcomes

In the validation set stratified by the TM frailty score, 27 (32.1%), 26 (31.0%), and 31 (37.7%) patients were in the fit, int-fit and frail subgroups, respectively. Grade ≥3 AEs were recorded for 7 (25.9%) fit, 14 (53.9%) int-fit, and 29 (93.6%) frail patients.

**Figure 4:**
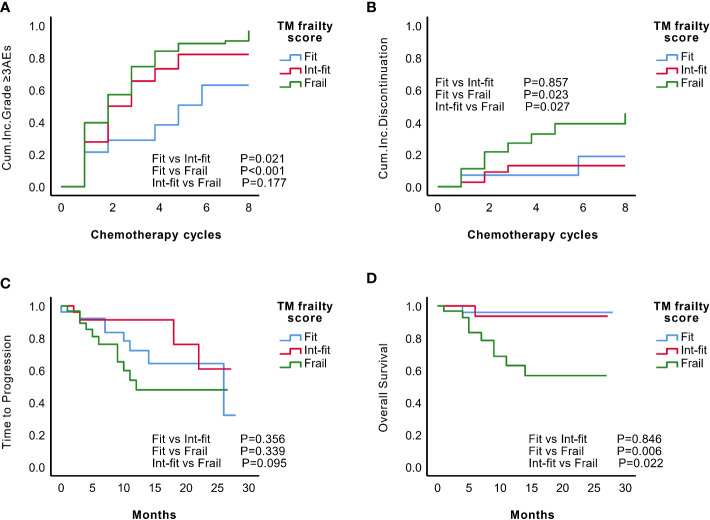
Predictive performance of the TM frailty score in the validation set (N=84). KM analysis of the occurrence of grade ≥3 AEs **(A)** and treatment discontinuation **(B)** according to the TM frailty scores of subgroups in the validation set. There was no significant difference in TTP **(C)** between the TM frailty score subgroups. The int-fit and fit subgroups showed better OS **(D)** than the frail subgroup.

The TM frailty score well distinguished the risk of grade ≥3 AEs in the fit and frail subgroups (log-rank test P<0.001; HR 2.03, 95% CI 1.02-4.03, P=0.002), as well as the fit and int-fit subgroups (log-rank test, P =0.021; HR 2.62, 95% CI 1.41-4.89, P=0.044) (**Figure 4A**, **Table 7**). The risk of grade ≥3 AEs was not well distinguished between the int-fit and frail subgroups (log-rank test, P =0.177). As shown in **Figure 4B**, the risk of treatment discontinuation was distinguished between the fit and frail subgroups (log-rank test, P=0.023) and between the int-fit and frail subgroups (log-rank test, P=0.027), but the statistical significance was lost in the multivariate analysis (p>0.05).

**Table 7:** Multivariate analysis of the impact of the TM frailty score in different datasets.

	Grade ≥3 AEs		Treatment discontinuation		TTP		OS	
	HR (95% CI)	*P*	HR (95% CI)	*P*	HR (95% CI)	*P*	HR (95% CI)	*P*
Fit	1	–	1	–	1	–	1	–
Int-fit	2.03 (1.02-4.03)	0.044	0.85 (0.22-4.45)	0.984	0.45 (0.13-1.59)	0.216	1.26 (0.79-20.2)	0.870
Frail	2.62 (1.41-4.89)	0.002	2.41 (0.70-8.32)	0.165	1.06 (0.37-3.02)	0.920	9.28 (1.17-73.33)	0.035

Adjusted for age and R-ISS stage.

The 1-year progression-free rate was 78.3% in the fit subgroup, 91.3% in the int-fit subgroup, and 47.9% in the frail subgroup. The 1-year OS rate was 95.7% in the fit subgroup, 93.3% in the int-fit subgroup, and 59.3% in the frail subgroup. No statistically significant difference in TTP between the TM frailty score subgroups was observed (P>0.05; **Figure 4C**). In the TM frailty score subgroups, OS was well distinguished between the fit and frail subgroups (log-rank test P=0.006; HR 9.28, 95% CI 1.17-73.33, P=0.035) and nearly significantly between the int-fit and frail subgroups (log-rank test P=0.022; HR 7.35, 95% CI 0.93-58.82, P=0.058). No difference was observed between the fit and int-fit subgroups (log-rank test P=0.846) (**Figure 4D**, **Table 7**).

#### 3.4.3 The ability of the TM frailty score to predict clinical outcomes

The C-index values of the TM frailty score for grade ≥3 AEs and OS were 0.741 and 0.702, respectively, showing much better predictive ability than the IMWG GA and IMWG GA PLUS (**Table 5**). The TM frailty score also helped identify the risk of treatment discontinuation (C-index=0.690, P<0.001). The TM frailty score could not identify the risk of TTP (C-Index=0.545, P=0.397).

## 4 Discussion

This study tested the IMWG GA to clarify its applicability in the Chinese MM population. In the IMWG GA subgroups, frail patients had a higher rate of grade ≥3 AEs, a higher rate of treatment discontinuation, and a greater risk of death than the fit subgroup. These findings indicate that the IMWG GA has clinical practical applicability to distinguish the risk of adverse clinical outcomes in Chinese MM patients. However, the IMWG GA was limited in that it could not distinguish the risk of grade ≥3 AEs or treatment discontinuation between the fit and int-fit subgroups. This suggests that the IMWG GA has limited applicability, so improvements were needed to achieve satisfactory risk stratification.

The reasons for the limited applicability of the IMWG GA are manifold. First, the age stratification in IMWG GA (≤75 years, 0 points; 76-80, 1 point; >80, 2 points) based on Western populations is not appropriate for Chinese MM populations. The median onset age reported in Chinese MM populations is 58 to 61 years (25–27), which is nearly 10 years younger than the onset age reported in European-American populations (69 years) (28). In our study, only 29.9% of the patients were aged 65 years or older, and only 6.59% of patients met the minimum age stratification criterion (>75 years) as defined by the IMWG GA. Second, chronological age is not a reliable indicator of the fitness status of older individuals. Even at the same age, the fitness status of individuals is significantly heterogeneous. Evaluation of the functional age by the CGA method can better obtain the actual fitness status of older individuals (3).

Third, the ADL and IADL, included in the IMWG GA, might contribute to its limited applicability. ADL only assesses the most basic activities of daily living, and it is limited in its ability to measure inconspicuous physical fitness decline. ADL is reported to have a significant floor effect (29). Only 2.1% of community-dwelling older individuals (>60 years) in China have ADL disability (30). In our study population, the ADL disability rate was 16.17%, while 31.1% of patients were IMWG GA frail. This finding indicated the limited contribution of ADL to identifying frailty. The IADL is reported to be biased by sex, education level, and economic status (31, 32). In the modern era, it has become easier for older people to carry out tasks such as laundry, shopping, and transportation, which will also affect the accuracy of IADL in assessing frailty. Given that functional status is the core element of the frailty assessment, better tools for evaluating performance status are desperately needed.

The CGA is a well-known multidimensional and multidisciplinary way to assess frailty status and the gold standard for identifying and managing frailty (11, 12). We performed the full CGA to find the CGA domains that were most valuable for improving the identification ability of IMWG GA. We found that the TUG and MNA-SF scores were independent factors related to grade ≥3 AEs and OS. TUG and MNA-SF were able to further stratify the risk of grade ≥3 AEs in the IMWG GA int-fit subgroups. In addition, MNA-SF risk-stratified patients in the IMWG GA-fit group. We believe that TUG and MNA-SF are good indicators to improve the discriminatory ability of IMWG GA.

Since nutritional status was not included in the IMWG GA, we added MNA-SF to the IMWG GA to develop the IMWG GA PLUS frailty score. Unlike IMWG GA, IMWG GA PLUS distinguished the risk of grade ≥3 AEs between its fit and int-fit subgroups. Regarding the ability to identify grade ≥3 AEs, the C-index of IMWG GA PLUS was improved from 0.662 (IMWG GA) to 0.701. This result indicated that the addition of MNA-SF slightly improves the ability to IMWG GA to identify chemotherapy tolerance. However, IMWG GA PLUS did not distinguish OS between its subgroups any better than IMWG GA did, and the C-index of IMWG GA PLUS was slightly lower than that of IMWG GA. In view of the fact that combining the MNA-SF does not improve the OS risk stratification capability, IMWG GA PLUS is not a satisfactory frailty score tool.

Given that TUG can better identify the chemotherapy tolerance and poor prognosis than ADL and IADL, we continued our exploration by adding the TUG score to the frailty assessment model to attain better identification. Instead of adding TUG to IMWG GA, we used TUG as a substitute for ADL and IADL because TUG, ADL and IADL are all tools for functional status assessment. The inclusion of overlapping tools increases the complexity and sacrifices the ease of use of frailty assessment tools. We used grade ≥3 AEs as the observational endpoint rather than OS, which was used in the development of the IMWG GA. OS is affected by frailty status and other, diverse factors, such as cytogenetic abnormalities, heterogeneity in sensitivity to treatment, and compliance levels. The longer the survival time is, the more confounding factors will appear. However, the original intention of evaluating frailty status was to predict the tolerance of patients to tailor a treatment plan appropriate for them. Finally, TUG, MNA-SF, and components of the IWMG GA were included in the multivariate Cox regression model. TUG and MNA-SF scores were the only independent risk factors for grade ≥3 AEs. Based on this result, we combined them as the TM frailty score. The TM frailty core ranged from 0 to 4 points and divided patients into 3 risk subgroups: fit (0), int-fit (1), and frail (2-4).

In the validation, the TM frailty score showed better identification ability for the risk stratification of grade ≥3 AEs and OS than the IMWG GA. First, the proportion of grade ≥3 AEs and deaths were higher in the TM frail subgroup than in the IMWG GA frail subgroup. Second, compared with the IMWG GA frail subgroup, the TM frail subgroup had a higher HR for grade ≥3 AEs (HR 2.62 vs. 1.57) and OS (HR=9.86 vs. 3.20), even though the proportions of the subgroups based on the two frailty assessment tools were similar. Third, the TM frailty score had higher C-index values than IWMG GA for grade ≥3 AEs (0.741 vs. 0.662) and OS (0.702 vs. 0.631). These results indicated that the TM frailty score had good discrimination ability and more precisely identified frail people than the more commonly used IMWG GA.

All of the scores are still limited by the fact that they cannot well distinguish the risks in their fit and frail subgroups from that in their int-fit subgroups, although the discriminatory ability of the TM frailty score for clinical outcomes was significantly improved. The TM int-fit subgroup was well distinguished from the fit subgroup, being comparable to the frail subgroup in the risk of grade ≥3 AEs, whereas in OS, the int-fit subgroup was well distinguished only from the frail subgroup, and its survival curve was similar to that of the fit subgroup (P<0.05). This indicated that although the chemotherapy tolerance of patients in the TM int-fit subgroup was poor, given that their OS was as good as that of the fit subgroup, adequate chemotherapy intensity is still required to ensure better long-term efficacy. Unlike the TM frailty score, the IMWG GA could not well distinguish OS between its int-fit and frail subgroups, even with the addition of the MNA-SF score. Therefore, the int-fit subgroup in the IMWG GA and IMWG GA PLUS should be treated as the frail subgroup, and adjusted treatment intensities are necessary for a better quality of life (33). In general, patients in the int-fit subgroup had unconventionality in their physical fitness and must be carefully evaluated to reduce missed diagnosis of the potential weakness. It is necessary to conduct a full CGA to determine their reversible health deficits, carry out clinical intervention to improve their treatment tolerance and achieve the upgrading of treatment goals.

There are some limitations to our study. First, our study is a single-center study with a small sample size and a short follow-up duration. In the validation of the IMWG GA, we found that there was no significantly difference in OS between the frail and fit subgroups. This nonsignificant difference may be due to the number of events and the short follow-up duration. Multicenter research with a larger sample and longer follow-up duration will help improve the generalizability of the results. In addition, the frailty status assessment is mainly used for older patients in clinical practice. The median age of our study population was only 59 years, and not enough older individuals were included. Therefore, this study must be continued, and the TM frailty score needs to be validated in a larger population of older adults to determine its clinical applicability.

## 5 Conclusion

The IMWG GA frailty assessment has limited applicability in the Chinese population. The TUG and MNA-SF domains were the most predictive CGA domains. The frailty-predictive capacity of the IMWG GA not satisfactorily improved by adding the MNA-SF score to it. Finally, TUG, MNA-SF, and the components of the IMWG GA were included to develop a novel frailty prediction model, namely, the TM frailty score, which reliably identified frail MM patients, showed better stratification of MM patients in terms of grade ≥3 AEs and OS than the IMWG GA and is thus more appropriate for directing treatment decisions in the Chinese population.

## Data availability statement

The original contributions presented in the study are included in the article/**Supplementary Material**. Further inquiries can be directed to the corresponding author.

## Ethics statement

Ethical approval was obtained from the ethics committee of the First Affiliated Hospital of Sun Yat-sen University. All patients provided informed consent.

## Author contributions

JLi, YC and JG designed the study. YC collected and analyzed the data and drafted the manuscript. JG directed the statistical analysis of the study and the review and critical revision of the manuscript. BH, JLiu and XL reviewed and modified the manuscript. JLi supervised the project and reviewed the research results and the manuscript. All authors approved the final version of the manuscript.
