# Supplementary material for: Development and validation of a chemotherapy tolerance prediction model for Chinese multiple myeloma patients: The TM frailty score
Source: Front Oncol. 2023 Jan 20;13:1103687. doi: 10.3389/fonc.2023.1103687 (PMC9895409; doi:10.3389/fonc.2023.1103687)
Supplement: Supplementary file 1 [file DataSheet_1.pdf]

Supplementary Table 1. Cases of grade ≥3 AEs

Toxicity Type			No. of times	Ratio (%)
Nonhematological AEs			187	76.02
	Infections			
		Pulmonary	78	31.71
		Uncertain position	21	8.54
		Herpes Zoster	18	7.32
		Skin and soft tissue	8	3.25
		Varicella	1	0.41
	Digestive system			
		Diarrhea	31	12.60
		Abdominal pain	3	1.22
		Liver damage	1	0.41
		Hypokalemia	3	1.22
		Gastrointestinal bleeding	1	0.41
	Cardiovascular			
		Atrial fibrillation	2	0.81
		Heart failure	5	2.03
		Myocardial injury	1	0.41
		Arrhythmias	1	0.41
	Neuromuscular system			
		Peripheral neuritis	2	0.81
		Muscle tremors	1	0.41
	Others			
		Death	4	1.63
		Rash	3	1.22
		Others	3	1.22
Hematological AEs			59	23.98
		Neutropenia	24	9.76
		Thrombocytopenia	34	13.82
		Lymphopenia	2	0.81

*Skin and soft tissue infections only recorded bacterial infections.

Supplementary Table 2. Multivariate analysis of the CGA domains for grade ≥3 AEs, treatment discontinuation, TTP,

CGA domain	**Grade ≥3 AEs**		**Treatment**		**TTP**		**OS**	
	**HR (95% CI)**	***P***	**HR (95% CI)**	***P***	**HR (95% CI)**	***P***	**HR (95% CI)**	***P***
TUG	1.39 (1.17-1.64)	**0.001**	1.02 (0.76-1.36)	0.920	1.08 (0.73-1.59)	0.701	2.11 (1.26-3.58)	**0.006**
CCI	1.19 (0.83-1.51)	0.467	1.07 (0.65-1.75)	0.807	1.02 (0.52-2.00)	0.950	1.20 (0.50-2.86)	0.686
MNA-SF	1.39 (1.14-1.67)	**0.001**	1.32 (0.97-1.80)	0.082	0.64 (0.40-1.02)	0.061	1.85 (1.01-3.40)	**0.046**
Polypharmacy	0.82 (0.60-1.14)	0.241	1.20 (0.71-2.05)	0.486	0.91 (0.73-1.13)	0.387	0.82 (0.61-1.11)	0.198
Mini-Cog	1.33 (0.65-1.84)	0.081	0.56 (0.32-1.00)	**0.049**	0.97 (0.65-1.47)	0.901	1.08 (0.61-1.91)	0.800
HADS	1.23 (0.90-1.66)	0.198	1.01 (0.58-1.73)	0.985	1.11 (0.58-2.12)	0.759	1.38 (0.57-3.30)	0.476
MOS-SSS	1.29 (0.95-1.74)	0.100	1.26 (0.75-2.13)	0.384	0.83 (0.44-1.55)	0.565	1.08 (0.46-2.60)	0.855
Geriatric syndromes	1.31 (0.95-1.80)	0.095	1.25 (0.72-2.17)	0.431	0.91 (0.43-1.94)	0.808	1.53 (0.61-3.85)	0.362

Adjusted for age and R-ISS stage

Supplementary Table 3. Comparison of the diagnostic value of functional status tools for AEs ≥ grade 3 and treatment discontinuation, disease progression, and death.

	AEs ≥ grade 3			Treatment discontinuation			Progression within 1 year			Death within 1 year		
	ΔAUC	*Z*	*P*	ΔAUC	*Z*	*P*	ΔAUC	*Z*	*P*	ΔAUC	*Z*	*P*
TUG vs. ADL	0.117	3.282	**0.001**	0.057	1.199	0.230	0.008	0.152	0.879	0.107	1.77	0.077
TUG vs. IADL	0.077	1.790	0.074	0.031	0.635	0.526	0.025	0.425	0.68	0.027	0.408	0.683
ADL+IADL vs. IADL	0.030	2.437	0.015	0.024	1.239	0.215	0.002	0.113	0.091	0.003	0.145	0.884
ADL+IADL vs. ADL	0.069	2.343	**0.019**	0.050	1.427	0.154	0.035	0.569	0.569	0.083	1.046	0.296
TUG vs. ADL+IADL	0.048	1.164	0.244	0.007	0.132	0.895	0.027	0.403	0.687	0.024	0.315	0.753

Supplementary Table 4. Cox multivariate analysis of the impact of the different functional status tools

	Grade ≥3 AEs		Treatment discontinuation		TTP		OS	
	HR (95% CI)	*P*	HR (95% CI)	*P*	HR (95% CI)	*P*	HR (95% CI)	*P*
TUG	1.39 (1.17-1.64)	**0.001**	1.02 (0.76-1.36)	0.920	1.08 (0.73-1.59)	0.701	2.11 (1.26-3.58)	**0.006**
ADL	1.53 (1.11-2.12)	**0.009**	1.18 (0.68-2.06)	0.559	1.29 (0.56-2.94)	0.549	1.28(0.43-3.79)	0.659
IADL	1.60 (1.20-2.13)	**0.001**	1.32 (0.82-2.13)	0.253	1.17 (0.63-2.18)	0.614	1.89 (0.77-4.67)	0.167

*Adjusted for age, R-ISS stage

Supplementary Table 5 Distribution of the clinical outcome in the TM frailty score in different datasets.

Dataset	Subgroup		Grade ≥3 AEs	Treatment discontinuation	Disease progression	Death
		N(%)	N(%)	N(%)	N(%)	N(%)
Entire Set（N=167）	Fit	52(31.14%)	18(34.62%)	9(17.31%)	15(28.85%)	3(5.77%)
	Int-fit	52(31.14%)	27(51.92%)	13(25.00%)	9(17.31%)	2(3.85%)
	Frail	63(37.72%)	54(85.71%)	34(53.97%)	18(28.57%)	17(26.98%)
Training Set（N=83）	Fit	25(30.12%)	11(44.00%)	6(24.00%)	7(28.00%)	2(8.00%)
	Int-fit	26(31.33%)	13(50.00%)	7(26.92%)	5(19.23%)	1(3.85%)
	Frail	32(38.55%)	25(78.13%)	13(40.63%)	7(21.88%)	8(25.00%)
Validation Set（N=84）	Fit	27(32.14%)	7(25.93%)	3(11.11%)	8(29.63%)	1(3.70%)
	Int-fit	26(30.95%)	14(53.85%)	6(23.08%)	4(15.38%)	1(3.85%)
	Frail	31(36.90%)	29(93.55%)	21(67.74%)	11(35.48%)	9(29.03%)
